# Is Attachment Theory the Answer to a Complex Healthcare System?

**DOI:** 10.1192/bjo.2022.337

**Published:** 2022-06-20

**Authors:** Alberto Salmoiraghi, Amy Kerti

**Affiliations:** 1 Betsi Cadwaladr University Health Board, Wrexham, United Kingdom; 2 Glwyndr University, Wrexham, United Kingdom

## Abstract

**Aims:**

This article proposes the need for a theoretical framework that can be applied to underpin the varied idiosyncratic mental health systems.

**Methods:**

Bowlby's Attachment Theory defines a set of values that are required for a developing child to acquire a stable base which allow for healthy psychological development into adulthood. These values and behaviours may serve as a caring and holistic framework for people using mental health services.

**Results:**

The outcomes in mental health remain unsatisfactory and services are overall fragmented and increasingly specialised. Ongoing recognition of the inter-related relationship between a person's immediate and social environment and their mental health are frequently overlooked as services become ever stretched in terms of finances, capacity and limited resources including support for staff. The emphasis of treatment is on illness instead of the multifactorial humanity of the individuals using the services. A key outcome of mental health provision is recovery but instead, recovery is compromised by a reductive approach to care that may paradoxically compromise rights, autonomy, confidence and self-belief when people are at their most vulnerable. This creates feelings of mistrust, uncertainty and a limited sense of safety toward services.

Attachment theory takes into account the individual, their experiences, their social world and the significant people in their lives. The principles required for the developing child to develop a secure attachment from a stable base are similar to those required for people experiencing mental illness to facilitate recovery and develop resilience to help manage and reduce episodes of relapse.

Systems that work well, frequently exhibit values underlying models of care that include continuity, consistency, respect, safety, autonomy, human rights, freedom, supportive, trusting relationships and collaboration. The opposite of that seen in what presents as autocratic and risk-averse approaches of many mental health services.

The principles required to enable a child to develop into a psychologically well-adjusted adult are similar to those required when a person is at their most vulnerable. Episodes of mental illness can be a time for reflection and growth, with the right care and therapeutic intervention, illness can also be a time to learn and develop skills for greater resilience in future.

**Conclusion:**

This paper outlines the implications and cultural changes that are required so that the principles of attachment theory can serve as a theoretical framework across mental health services to provide a stable base for people using the services and staff providing the care.

